# The rise of rapid implementation: a worked example of solving an existing problem with a new method by combining concept analysis with a systematic integrative review

**DOI:** 10.1186/s12913-020-05289-0

**Published:** 2020-05-21

**Authors:** James Smith, Frances Rapport, Tracey A. O’Brien, Stephanie Smith, Vanessa J. Tyrrell, Emily V. A. Mould, Janet C. Long, Hossai Gul, Jeremy Cullis, Jeffrey Braithwaite

**Affiliations:** 1 Centre for Healthcare Resilience and Implementation Science, Australian Institute for Health Innovation, Macquarie University, Level 6, 75 Talavera Road, North Ryde, NSW 2109 Australia; 2 Faculty of Medicine, School of Women’s and Children’s Health, University of New South Wales, Sydney, NSW Australia; 3 Kids Cancer Centre, Sydney Children’s Hospital, Randwick, Sydney, Australia; 4 Nuffield Department of Orthopaedics, Rheumatology & Musculoskeletal Sciences, Botnar Research Centre, University of Oxford, Oxford, UK; 5 School of Nursing and Midwifery, Edith Cowan University, 270 Joondalup Drive, Joondalup, WA 6027 Australia; 6 Perth Children’s Hospital, Nedlands, Perth, Australia; 7 Lowy Cancer Research Centre, Children’s Cancer Institute, University of New South Wales, C25/9 High Street, University of New South Wales, Kensington NSW, Sydney, 2750 Australia; 8 Clinical librarian, Information Access and Advisory Services, Macquarie University Library, Macquarie University, 16 Macquarie Walk, North Ryde, NSW 2109 Australia

**Keywords:** Concept analysis, Implementation, Implementation science, Healthcare, Rapid implementation, Systematic integrative review

## Abstract

**Background:**

The concept of rapid implementation has emerged in the literature recently, but without a precise definition. Further exploration is required to distinguish the concept’s unique meanings and significance from the perspective of implementation science. The study clarifies the concept of rapid implementation and identifies its attributes, antecedents, and consequences. We present a theoretical definition of rapid implementation to clarify its unique meaning and characteristics.

**Methods:**

Rodgers evolutionary concept analysis method, combined with a systematic integrative review, were used to clarify the concept of rapid implementation. A comprehensive search of four databases, including EMBASE, MEDLINE, SCOPUS, and WEB OF SCIENCE was conducted, as well as relevant journals and reference lists of retrieved studies. After searching databases, 2442 papers were identified from 1963 to 2019; 24 articles were found to fit the inclusion criteria to capture data on rapid implementation from across healthcare settings in four countries. Data analysis was carried out using descriptive thematic analysis.

**Results:**

The results locate the introduction of rapid implementation, informed by implementation science. Guidance for further conceptualisation to bridge the gap between research and practice and redefine rigour, adapting methods used (current approaches, procedures and frameworks), and challenging clinical trial design (efficacy-effectiveness-implementation pipeline) is provided.

**Conclusions:**

It is possible that we are on the cusp of a paradigm shift within implementation brought about by the need for faster results into practice and policy. Researchers can benefit from a deeper understanding of the rapid implementation concept to guide future implementation of rapid actionable results in clinical practice.

## Background

Implementation may be broadly defined as putting an intervention into effect when delivered in a setting, and is one critical element of evidence-based practice [1]. Implementation science is the rigorous study of implementation, described as the method to promote the uptake of clinical research findings and other evidence-based practice into routine practice and hence improve the quality and effectiveness of healthcare [2]. Unfortunately, these definitions exclude a temporal aspect – that is, how we get what works to the people who need it with the greatest speed and efficiency. Some of the early developers of implementation science recognised this in real-world systems-thinking and methods and began responding to estimates that the time it takes to implement research into clinical practice is 17 years on average, with low uptake of evidence-based findings implemented in practice and poor effect sizes when adopted [3]. This time-gap paradigm has created many challenges for practitioners and policy makers who need rapid, actionable results, such that multiple stakeholders (e.g., practitioners, patients, families, decision-makers, administrator and policy makers) are beginning to question implementation success [4, 5]. This is understandable given the poor outcomes from these necessary but what seems insufficient approaches. There remains a troubling implementation gap, defined as the difference between our knowledge of what works and the time it takes to get that knowledge into practice in real-world settings.

Rapid implementation is an intriguing possibility to narrow the implementation gap. We can ask whether rapid implementation can be informed by implementation science, but it has yet to be defined in the literature, and studies are few and far between; nor has there been a systematic review on rapid implementation studies undertaken to date within the healthcare and medical spheres. This absence impedes our ability to understand and enable rapid, evidence-based findings to find their way quickly into clinical practice [6]. Similarly, the importance of defining concepts has been shown in the work of behaviour change interventions indicating that without standardised behavioural definitions it is difficult to replicate effective interventions and challenging to identify techniques contributing to effectiveness across interventions [7]. By providing a clear definition of rapid implementation, we avoid concerns previously directed at the science and practice of implementation related to poor consistency of terminology for core concepts that resulted in researchers characterising implementation science as a Tower of Babel [8]. A clear definition will ensure that throughout the research or implementation science field we are all talking about rapid implementation in the same way. This will aid the research community to communicate effectively within and between disciplines, and to apply evidence-based research findings [9]. We sought to use concept analysis to provide a theoretical definition and identify essential elements of rapid implementation.

Triangulation of methods has been argued to be the future of implementation science— enhancing understanding of data findings, and as a result, shining a light on research challenges from multiple perspectives [10]. No one method reveals absolute truth or provides a definitive standpoint [11]. A new method combination, concept analysis and systematic integrative review, is introduced in this paper, for the first time to our knowledge, having only been used separately in previous research [12–14].

Rapid implementation has a possibility to narrow the evidence-practice gap by addressing the delay of implementing research into practice and is an entirely new concept. Concept analysis is a method for clarifying foundational ideas and is derived from a deep analysis of core elements of a target problem or issue under investigation [15–17]. By way of contrast, an integrative review provides a systematic approach to data examination, and considers a range of diverse studies, often traversing both qualitative and quantitative methods, with synthesis and conclusions drawn.

The decision to triangulate concept analysis and integrative review led to the formulation of two aims for the present study: 1) provide an understanding and definition of rapid implementation, informed by knowledge drawn from the implementation science field, and 2) demonstrate the contributions of concept analysis and integrative review, conjoining the strengths of each through this worked example.

## Methods

The purpose of a concept analysis is to analyse, define, develop and evaluate ambiguous or complex concepts [18] and provide a precise definition. A number of methods have been developed to guide the analysis of a concept [19]. In a recent scoping review of concept analyses by Rodgers [20], the Wilson Method was the most commonly used (Walker & Avant, [21]; *n* = 465), followed by the evolutionary Method (Rodgers [22]; *n* = 213) and then the Principle-based Method (Morse et al. [23]; *n* = 47). Rodgers [20] also highlighted a possible lack of rigour, restricted scope, and failure to approach conceptual work in a systematic way in many of the papers analysed. The Wilson Method [21] has been suggested to enhance critical thinking but has been critiqued for not necessarily producing documentation of a scientific nature [24]. Yet the Principle-based Method [24] has been praised for its robust means of theoretically defining a concept and determining the state of science at any given point in time [25], whilst we found the guidelines to do this to be rather indistinct. We selected Rodgers [26] Evolutionary Concept Analysis Method because of the emphasis it gives to the examination of the quality and the degree of the concept reported in the literature. Rodgers’ [26] traditional step-by-step linear approach can be limiting, compared to a fluid three-phase evolutionary concept analysis approach, previously described by Tofthagen and Fagerstrøm [27] and Delves-Yates, Stockl [18] as consisting of: Phase 1 Initial phase; Phase 2 Core analysis; and Phase 3 Further analysis. Combining the three-phase evolutionary method of concept analysis with a systematic integrative review provides an organized process that may enhance rigour, with the systematic integrative review addressing both qualitative and quantitative studies, and enabling a more thorough, integrative review of papers covering a range of methodologies [12]. This attempt to produce a more robust and transparent process of assessing the concept of rapid implementation within the literature may lead to more useful and relevant definitions of a concept [20], with the literature in this case being used as the primary source of data [28]. Table 1 highlights how components of the phases and stages of evolutionary concept analysis were augmented by additional stages for conducting a systematic integrative review.

**Table 1:** Proposing an augmented three-phase framework that combines concept analysis with a systematic integrative review

Phase	Stage 1	Stage 2	Stage 3	Stage 4
1: Initial phase	Identify and name concept	Identify and select an appropriate sample for data collection	Identify surrogate terms and relevant uses of the concept and describe the search strategy	Identify databases searched, inclusion/exclusion criteria, data extraction, methodological quality, and synthesis
2: Results & Core Analysis	Study characteristics and risk of bias (quality) assessment	Identify the attributes references, antecedents and consequences of the concept	Identify concepts related to the concept of interest	Identify a model case of the concept
3: Further Analysis	Further development of the concept	Strengths and limitations		

We also applied an adapted version to that of Whittemore and Knafl’s [29] systematic integrative review, with a previously adapted version applied to nursing [12] and intrapersonal, interpersonal and structural barriers in goal-driven behaviours [13]. This adaption created an additional step of appraisal of the overall quality of each paper mentioning rapid implementation. This in turn allowed for: 1) description of the search strategy, 2) identification of databases searched, 3) listing inclusion/exclusion criteria, 4) data extraction 5) methodological quality, and 6) synthesis.

### Phase 1 initial phase

#### Phase 1; stage 1: identify and name concept

The concept ‘rapid implementation’ was selected, having emerged in the literature recently, but without a precise definition.

#### Phase 1; stage 2: identify and select an appropriate sample for data collection

Whittemore and Knafl’s [29] systematic integrative review method was adopted for this stage. No reviews on implementation science were identified in the Database of Abstracts of Reviews of Effects (DARE) and the Cochrane Database of Systematic Reviews (CDSR). A protocol was developed that included review questions, inclusion and exclusion criteria, search strategy, study selection, data extraction, quality assessment, data synthesis, and plan for dissemination [30].

#### Phase 1; stage 3: identify surrogate terms and relevant uses of the concept and describe the search strategy

The surrogate terms helped form the key terms used for the search strategy alongside and guided by PICO, for example, the use of P = population, I = phenomena of interest, Co = context (P = the delay in implementing research into practice, I = rapid implementation of research, CO = the Hospital/Healthcare setting). Final search terms were reviewed by an experienced clinical librarian (JC) for the Centre for Healthcare Resilience and Implementation Science, who consulted on the search strategy and databases to use. A variation of the following key terms was searched in various combinations:

1. (“rapid research” or “rapid implementation”).Mp
2. implementation science/.
3. implementation science.mp.
4. (dissemination or implementation).Mp
5. implementation research.ti,ab.
6. 2 or 3 or 4 or 5.
7. 1 and 6.

#### Phase 1; stage 4: identify databases searched, inclusion/exclusion criteria, data extraction, methodological quality, and synthesis

**Fig. 1:**
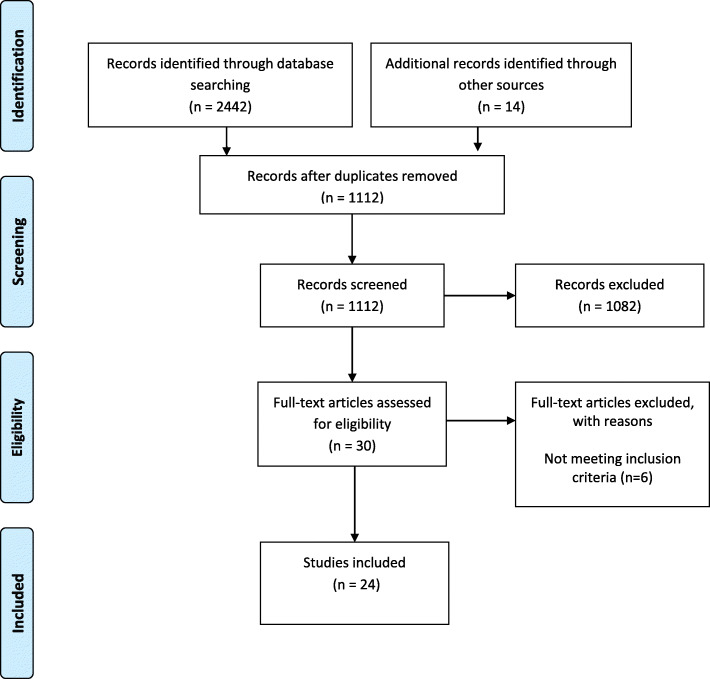
PRISMA flow diagram of the screened studies

The description of the databases and inclusion/exclusion criteria are shown in Table 2. Additional articles meeting the inclusion criteria were obtained through hand searching of relevant journals (see Fig. 1). Also, recognition of references for inclusion occurred when sources were cited frequently by other authors but had not been identified in the original search results (snowballing).

**Table 2:** Inclusion and exclusion criteria and databases

Inclusion	Exclusion	Database	Total
Primary data-based studies (not excluding literature reviews)	Reports, conferences or discussions, including unpublished manuscripts, books, tapes, and electronic media	EMBASE	382
Studies have to be explicit to ‘rapid implementation’ within implementation science	Publications were excluded if they were non-English articles	MEDLINE	380
Studies were only included if they specify a hospital or health related context		SCOPUS	996
Studies included regardless of methodology		WEB OF SCIENCE	684

* Language limiter was set to English language for the database search

#### Data extraction

Figure 1 provides the study flow diagram using Preferred Reporting Items for Systematic Reviews and Meta Analyses (PRISMA) that provides the number of articles and duplicates removed and the process of selecting the final studies. JS and SS performed the study selection by reading the title and abstracts of all studies and sequentially excluded records according to the inclusion/exclusion criteria. If the title and abstract met the inclusion criteria the full text of the articles was read to determine if inclusion criteria were met. Data relating to rapid implementation were extracted after each publication was read line-by-line. To add to the rigour of this process, the data extraction process was then repeated independently by JC who checked the process to ensure credibility and reduce personal bias.

#### Methodological quality (risk of bias, quality)

To facilitate inter-rater agreement, a rating format for both qualitative and quantitative studies was used. The Standard Quality Assessment Criteria for Evaluating Primary Research Papers (QualSyst) criteria we adopted was set out by Kmet et al. [31] closely followed by a more recent systematic review [32] that we used in conjunction with the PRISMA reporting guidelines. Inter-rater agreement was assessed using percent agreement (number of agreement scores divide by the total number of scores) [32].

Two authors (JS, SS) assessed methodological quality using the Standard Quality Assessment Criteria for Evaluating Primary Research Papers (QualSyst) for eligible articles [31, 32]. Quantitative studies were scored on 14 criteria, while qualitative studies were scored on 10 criteria (see Tables 3 and 4). Mixed-methods papers were scored on both criteria. Each article was given a score of 0 (not met), 1 (partially met), or 2 (met) for each criterion [31, 32]. A summary score was calculated for each study by summing scores for each criterion and dividing the total possible score, where higher scores indicated greater methodological quality [32]. Agreement for these studies was 89%. Disagreements were resolved through discussion until consensus was reached.

**Table 3:** Percentage of studies scoring ‘Yes’ for quality assessment criteria (Kmet et al. [31]; Collins et al. [32])

Criteria		n	%
**Qualitative Criteria**			
1	Question/objective sufficiently described?	21	100
2	Study design evident and appropriate?	21	100
3	Context for the study clear?	20	95
4	Connection to a theoretical framework / wider body of knowledge?	21	100
5	Sampling strategy described, relevant and justified?	4	19
6	Data collection methods clearly described and systematic?	4	19
7	Data analysis clearly described and systematic?	4	19
8	Use of verification procedure(s) to establish credibility?	3	14
9	Conclusions supported by the results?	19	90
10	Reflexivity of the account?	6	29
**Quantitative Criteria**			
1	Question / objective sufficiently described?	4	100
2	Study design evident and appropriate?	4	100
3	Method of subject/comparison group selection or source of information/input variables described and appropriate?	4	100
4	Subject (and comparison group, if applicable) characteristics sufficiently described?	N/A	N/A
5	If interventional and random allocation was possible, was it described?	N/A	N/A
6	If interventional and blinding of investigators was possible, was it reported?	N/A	N/A
7	If interventional and blinding of subjects was possible, was it reported?	N/A	N/A
8	Outcome and (if applicable) exposure measure(s) well defined and robust to measurement / misclassification bias? Means of assessment reported?	3	100
9	Sample size appropriate?	4	100
10	Analytic methods described/justified and appropriate?	4	100
11	Some estimate of variance is reported for the main results?	4	100
12	Controlled for confounding?	4	100
13	Results reported in sufficient detail?	4	100
14	Conclusions supported by the results?	4	100

Note. Not all criteria were applicable for all studies; QAT = Quality Assessment Tool

**Table 4:** Summary of the included studies

No	References	Date	Country	Type	Aim	Context	Outcome	QAT summary score
1	Bando	2017	Japan	Literature review	To review precision medicine in Japan and Europe	Oncology and precision medicine	Barriers faced in rapid implementation of precision medicine are apparent in Japan, and further effort and collaboration will be needed for Japan to take a lead in establishing precision medicine	50
2	Basu	2013	America	Quantitative	To provide a more efficient and powerful tool to perform gene-based genome-wide association study with single or multivariate traits	Bio statistics and precision medicine	The authors propose a new approach for rapid implementation for gene-based genome-wide association studies	100
3	Battaglia	2018	America	Literature Review	To discuss pragmatic models, methods, and measures in implementation	Nursing research and enhancing population health	Pragmatic dissemination and implementation approaches are needed to speed up research translation	50
4	Beck	2009	America	Mixed-method	The use of a conceptual framework for implementation and dissemination	Child Care and paediatric practice at a Health Maintenance Organization	Rapid implementation is seen through combining implementation frameworks (PRISM & RE-AIM) and augmenting components with social network analysis	Qual: 90 Quant: 100
5	Bernstein	2009	America	Quantitative	To increase the use of smokers’ quitline referral services	Smoking referral Quitline	Marked and sustained use of quitline referral services by health care providers	100
6	Birenda	2015	America	Quantitative	To provide further data to delineate the progression free survival of patients who get treated with targeted therapy in molecular profiling	Cancer	Demonstrates the potential value of molecular profiling. Continued work on rapid implementation of molecular profiling earlier in the care of oncology patients continue to be a future goal	100
7	Burkard	2017	America	Observational	Introducing a state-wide molecular tumor board	Community oncology practices	The molecular tumor board approach provides flexibility and rapid implementation by integrating clinical service, a registry, and a journal club	40
8	Churruca	2019	Australia	Multi-case analysis	Researchers and implementers working together in situ throughout an implementation project	Adapting Implementation Approaches	Embedded implementation research approaches hold promise for rapid implementation	50
9	Denomme	2008	Germany	Commentary	Dry matching to improve transfusion outcomes for widespread implementation by rapid timelines through standards of practice	Blood banks	Discusses rapid implementation of donor-recipient blood group genotype dry-matching would have on reducing the incidence of delayed transfusion reactions and its associated comorbidities	50
10	Francescatto	2015	America	Literature review	Precision medicine and speedy implementation in the clinical setting	New born screening	Discusses rapid implementation through to the possibility of having complete access to our genetic data from birth, if not shortly after conception	40
11	Gale	2019	America	Qualitative	Comparison of rapid transcription procedures	Opioid prescribing in the Veterans Health Administration	Rapid analyses is on the rise providing valid findings in a short timeframe, enabling identification of actionable recommendations	100
12	Glasgow [1]	2014	America	Literature Review	To provide lessons learned from the My Own Health Report Project	Primary care	Conducting complex studies rapidly and efficiently is a realistic goal	50
13	Glasgow [2]	2003	America	Literature Review	To discuss the efficacy-effectiveness trials	Rapid implementation within healthcare	Recommend key conceptual and methodological characteristics are offered to help close the gap	50
14	Glasgow [3]	2012	America	Literature Review	To determine what is needed for rapidly integrating science into practice	Rapid implementation within Health care	Different approaches are needed for rapid robust sustainable real world healthcare programs and policies. To produce different outcomes, we need to think and act differently	50
15	Glasgow [4]	2012	America	Literature Review	To address the gap between current knowledge and practice in the area of dissemination and implementation research	Rapid implementation within healthcare	Further advances in the field will be achieved by focusing dissemination and implementation to become more rapid	50
16	Guthrie	2014	UK	Case Study	Investigating time lags from research to practice	Public charitable investment in cancer research	Having networks in place can support rapid research translation	50
17	Keith	2017	America	Qualitative	Intervention-specific codes, and CFIR constructs to reduce and organize the data to speed up procedures and analysis	Primary care practices	Using the CFIR to guide data collection, coding, analysis, and reporting of findings supported a systematic, comprehensive, and timely understanding of barriers and facilitators to practice transformation	100
18	Kilbourne	2017	America	Literature Review	Using QUERI to support rapid implementation into clinical practice	Rapid implementation within healthcare	Shows how to rapidly translate research findings or evidence-based treatments (best practices) into clinical practice	100
19	Krier	2016	America	Literature Review	To discuss precision medicine applications, challenges and opportunities	Genomic sequencing in clinical practice	Discusses clinical innovation, rapid implementation and complicated implementation questions	50
20	Peek	2014	America	Literature Review	To discuss different approaches to make health care research more relevant and rapid	Rapid implementation within healthcare	Emerging standard of research 5 R’s	50
21	Rapport [1]	2018	Australia	Literature Review	Predicting a new approach to methods	Rapid implementation within healthcare	For rapid implementation we need new methods	50
22	Rapport [2]	2018	Australia	Literature Review	The authors aim to reveal how implementation science is presented and understood in health services to progress our knowledge	Rapid implementation within healthcare	Rapid implementation is about adaption. Implementation science models, theories, and frameworks are critiqued.	50
23	Reeves	2013	America	Literature Review	Exploring ethnography	Rapid ethnography as a method to study healthcare	Rapid ethnography reduces the time spent in observation when compared to traditional ethnography	60
24	Riley	2013	America	Literature Review	Speed up research into practice	Rapid implementation within healthcare	Proposing rapid learning systems to evaluate new and existing treatments	50

Note.  QAT = Quality Assessment Tool. Part of the inclusion criteria was not to exclude literature reviews because rapid implementation is a new area, therefore, it was decided that valuable data may be lost had these studies been excluded. However, the assessment of literature reviews meant that not all of the criteria were applicable thus lowering assessment scores for these specific studies on the QAT

#### Synthesis

Systematic integrative reviews help bring data together in a descriptive thematic synthesis [13, 33]. For data evaluation, studies were reviewed, categorised and critiqued [34]. NVivo v12 plus [35], a data management tool to facilitate both the synthesis and critique process was used.

#### Analysis

Data analysis was carried out using descriptive thematic analysis adapted from Thomas et al. [33]. Articles were read and reread and text reviewed line by line, to obtain a detailed understanding and familiarisation. Descriptive thematic analysis with iterative processes created the themes using the following approach. Significant information from the studies were coded and sub-categorised and classified into attributes, antecedents, and consequences. Once classified, the codes were reviewed for overarching themes as outlined in Table 5. JS led the analysis and consensus was reached during team meetings, where findings were critically examined and questioned by all authors.

**Table 5:** Themes developed from a synthesis of the literature

Antecedents	Attributes	Consequence
Precision medicine (Molecular immunohaematology, Molecular Tumour Boards & Genotyping)	Redefining rigor	Research and Practice
	Rapid-Learning research system	Re-thinking trials
	Adapting implementation frameworks for use within rapid implementation	
	Tailoring methods and approaches	

## Results

### Phase 2: results and core analysis

#### Phase 2; stage 1: study characteristics and risk of bias (quality) assessment

##### Study characteristics

Our derived sample consisted of 24 studies selected for the period of 2003–2019: 18 studies were from the United States of America (America from here), three were from Australia, one was from Japan, one from Germany and one from the United Kingdom (U.K. from here). Authors such as Glasgow [11, 36–40] from America, and Braithwaite and Rapport [10, 41, 42] from Australia, frequently appeared in the literature. Some areas of clinical practice were prominent in calling for rapid research to align with the speed of progress, for example, precision medicine [43–47]. A diverse range of methodological approaches were taken, including qualitative, quantitative and mixed-methods.

##### Risk of bias (quality) assessment

Table 3 shows that the quality of studies was generally high with quality assessment scores ranging from 40 to 100% (mean QAT score is 100% for quantitative, and 59% for qualitative) confirming the findings of a recent study using the same assessment tool [32]. Although scores for the quantitative studies were high, this was not the case for qualitative studies. For example, part of the selection criteria involved the inclusion of literature reviews to enrich the information available for assessment; however, not all criteria were applicable for assessing literature reviews thus lowering quality assessment for these studies (see Table 4). While quality assessment was not a criterion for inclusion, in line with Sandelowski, et al’s [48]. study, to exclude studies based on quality appraisal could result in valuable data being lost. Instead, quality appraisal processes were used to both increase familiarity with the studies and highlight methodological rigour of studies.

##### Core analysis

Themes that were derived from the synthesis of the studies have been classified under attributes, antecedents and consequence, as shown in Table 5. Antecedents of rapid implementation included clinical practice areas e.g., precision medicine. Attributes of rapid implementation included adaptions to methods (current approaches, procedures and frameworks). The consequence is to bridge the gap between research and practice by re-thinking trials to produce more speedy actionable data that can be of use to practitioners and people working in the field of healthcare delivery.

#### Phase 2; stage 2: identify the attributes, references, antecedents, and consequences of the concept 

##### Attributes

Attributes are characteristics of the concept that make it possible to identify situations that can be characterised under the concept and constitute the essential definition of a concept [27, 49]. The defining attributes of the term rapid implementation include rapid [37, 39, 44, 50], responsive [10, 39, 51], relevant [37, 42, 52], efficient [36, 37, 44], and speedy [4, 37, 39] research findings that are produced because of more flexible designs (methods [10, 44, 53], approaches [42], procedures [37, 50], and implementation science frameworks [40, 50, 54]. Other attributes include calls to re-define research rigour [11, 37, 41, 52] which entails promoting research that is both thorough, relevant and that disseminates well into practice to increase the timeliness and applicability of research. Increasing the timeliness and applicability of research innovation, and establishing rapid learning research systems [4, 42, 51] which are considered to bring researchers, funders, practitioners, and those working in health systems together to assist in the rapid adoption of research findings in practice.

##### Antecedents

In consideration of these defining attributes, antecedents are events preceding the concept [26, 49]. The antecedents for rapid implementation are clinical practice antecedents e.g., precision medicine [43–47, 55], and are being viewed across the biomedical enterprise, such as molecular immunohaematology (molecular oncology) [46], molecular profiling (oncology) [45], molecular tumour boards (precision oncology) [55], and genotyping (biostatistics) [44]. These are rapidly evolving areas that require rapid deployment of actionable data. It appears that these specific clinical areas are indeed driving the concept of rapid implementation in clinical practice.

##### Consequence

Consequences are defined as events or phenomena that result from the concept [26, 49]. The consequence of rapid implementation requires change to traditional study designs that are notoriously slow to change, with pipeline problems (efficacy, effectiveness and implementation). This includes calls for more appropriate trial designs such as basket trials (discovery-based, which can be phase I or early phase II trials), umbrella trials [43] (which can be phase II, exploratory, or proof-of-concept trials) and qualitative trials [10] all of which attempt to bridge the research to practice gap. Consequences, therefore, reflect what we know works and how to get it into practice faster, to respond to questions of practitioners or decision-makers who make decisions about health care, and who need rapid, actionable data to make those decisions.

#### Phase 2; stage 3: identify concepts related to the concept of interest

Rapid implementation is successful when results are used widely across healthcare settings. As a science area, precision medicine is, for example, changing the way we practice medicine and deliver healthcare by calling for faster, actionable results, and timelines to be shortened, from discovery and application in laboratories, to their recognition as standards of practice [46].

#### Phase 2; stage 4: identify a model case of the concept

The following defining criteria of rapid implementation is presented in an identified model case. It is intended to illustrate and help understand rapid implementation in use.

Developments in next generation sequencing and information technology have made precision medicine possible, with genetic, omics, clinical, environmental and lifestyle data now available [43]. Scientific and technological advances occur that may make ‘business as usual’ less relevant or even obsolete. Precision medicine is a disruptive innovation that holds the potential to fundamentally alter how evidence-based medicine is practiced and taught (Rushforth A, Greenhalgh T: Personalised medicine, disruptive innovation and ‘trailblazer’ guidelines: Case study and theorization of an unsuccessful change effort, forthcoming). This is at the core of what is driving real time translation at a different speed. Thus, the gulf between research and practice is affecting clinicians who need rapid, actionable data to make decisions. Acquiring research in more rapid ways suggests that practice questions could shape the research methods used, rather than the methods determining the research agenda. Rapid deployment of results means we need to redefine rigour and provide a degree of flexibility.

#### Proposed theoretical definition

The intent of the proposed theoretical definition is to highlight how the analysis revealed rapid implementation as a key concept. The findings indicated no clear theoretical definition at present. Drawing on our analysis we propose the following theoretical definition for rapid implementation:

*Rapid implementation provides the best possible evidence-based practice of a program or intervention to those who need it, with speed and efficiency, by redefining rigour, and adapting both methods (adapting current approaches, procedures and implementation frameworks), and trial design, to fit research aims and objectives.*

## Discussion

### Phase 3; stage 1: further development of the concept

The work in this paper provides a method to increase our understanding of rapid implementation in terms of doing all types of implementation science more efficiently, with rapid implementation as an intriguing possibility to bridge the gap between research and practice and get actionable results into practice more quickly and effectively. We attempted to uncover the core concepts in the literature and synthesise the findings from papers defining themselves as involved in some respect in a rapid implementation, within the broad remit of implementation science. The concept of rapid implementation until now has been without a precise definition. The result of the study leads to a precise definition, derived from establishing the meaning, attributes, and characteristics of rapid implementation.

The theoretical definition derived from our results characterises rapid implementation as incorporating speed and efficiency, while having the ability to adapt methods and trial design to suit the needs of complex studies. The literature in this area is still in its infancy and remains largely descriptive in terms of how study design and strategies can reduce the time it takes to move evidence into practice. Our study has brought this to light, by focusing on defining rapid implementation as an emerging area of importance, and by so doing, providing a fundamental definition (‘building blocks’) of rapid implementation that is for the first time being made explicit. This not only ensures the international community can communicate more effectively within and between disciplines [9], but that research results have the potential to be more valid and reliable. Having a standard definition of rapid implementation may make it possible for research to replicate effective interventions [7] and shape future research to improve the evidence-base.

### Links across themes

The fundamental basis of rapid implementation is clinical practice – with its need for fast information on which to base good clinical decisions. It is exemplified here by precision medicine, which is amongst those areas leading the field in the application of rapid implementation approaches and ideas [43–46, 55]. Attributes of rapid implementation (adapting methods, procedures, and frameworks) are challenging traditional implementation and the consequence is that rapid implementation can help bridge time-gaps between research and practice (working on, for example, research practice and clinical policy simultaneously, or re-thinking and shortening the length of trials). In particular, antecedents that relate to clinical practice areas and attributes of rapid implementation that challenge traditions are of particular significance, appreciating the bidirectional relationship between practice and research. The clinical practice studies retrieved discussed science areas such as genomics that have evolved quickly within the precision medicine paradigm [43–47, 55] and suggested that these areas require research findings to be produced quickly, to provide recommendations, so that a patient can be treated in a timely way. These efforts have now included calls for more appropriate trial designs such as basket trials or umbrella trials [43] and qualitative trials [10]. Ways to address this problem can be found within the core attributes of rapid implementation: to feedback information and findings more quickly to clinical practice. The research literature also calls for a redefinition of rigour in undertaking a rapid implementation study as part of the implementation science agenda, along with the view that rapid learning research systems need to be deployed to ensure that research can meet the time-pressured demands of clinical practice [56]. In this respect, there is broad agreement amongst researchers and practitioners that there needs to be a common cause to support the rapid implementation of research findings into clinical practice. Harnessing rapid learning research systems and precision medicine models of care together may foster greater stakeholder collaborations, encouraging greater integration between researchers, funders, health systems workers, practitioners, and community partners, focussing on time-pressured, clinically relevant questions [39].

### Antecedents of clinical practice areas

Clinical practice areas within the broad remit of precision medicine [43], such as molecular oncology [46], molecular profiling [45], molecular tumour boards [55], and genotyping [44] all require rapid implementation, creating urgency for implementation science to research the most effective ways to inform how we create those changes. Churruca et al. [42] discussed genomics and the role of evidence within implementation science, highlighting why evidence slowly and only inconsistently makes its way into practice [3]. In support, Peek et al. [52] argued that it was unacceptable that only 14% of funded research made its way through the minefields of uptake into practice [3]. Putting this into perspective, precision medicine’s emerging technologies have evolved so much in the time it takes to implement change in real-time practice that the original protocols developed ahead of the subsequent research findings can be redundant.

### Attributes at the core of rapid implementation

Research challenging traditional implementation is essentially about research being more responsive. We need to transition from traditional implementation towards more sustainable, rapid implementation. Rapid research must provide actionable results and scientific rigour, discussed by Rapport and Braithwaite [10] and Peek et al. [52]. To uphold rigour, we need to redefine it to reflect the needs of a range of stakeholders (for example; practitioners, decision-makers, and policy makers), to reflect a more pragmatic approach to research. Peek et al. [52] suggest current conceptions of rigour do not allow for this and limit the range of real-world situations where intervention implementation is feasible. Striking a balance between rigour, rapidity and flexibility of methods and procedures [55] is difficult however to achieve [37, 50].

In redefining rigour, we must be mindful that research aims and objectives should determine the research methods rather than the methods driving the research agenda [57]. If contexts and needs require rapid implementation, then current methods must be adapted [58]. To help understand the mechanisms and contexts of implementation, researchers are exploring generating speedy actionable findings through mobile methods [10, 59], case studies [42, 53], and the transition from traditional ethnographic methods to rapid ethnography [60] to inform rapid improvements to healthcare. These initiatives are part of the overarching shift towards rapid implementation science by researching the most efficient ways to implement evidence [52].

We must also reassess how we manage data. Some studies have introduced ‘rapid analysis’ [50], defined as adapting procedures to produce speedy, efficient and valid findings, as well as providing timely information of value to stakeholders (practitioners, patients, families, decision-makers, administrator and policy makers) [37]. This may also mean adjusting recruitment processes and survey procedures to enhance participation rates [50].

A structural mechanism for progress is becoming known as ‘rapid learning research systems’ [39]. Churruca et al. [42] presented case studies that explored a rapid learning research system in the field of genomics and suggested a new approach recommending that implementation scientists be embedded within the very fabric of the healthcare system with the implementation scientist being viewed as one of the team. Guthrie et al. [53] presented case studies showing a number of different actors (practitioners, surgeons, policy makers) playing a role in bridging the gap between research and practice. For Churruca et al. [42] this can build social capital by sharing knowledge with, for example, local clinical and laboratory genomics researchers. In rapid learning research systems, stakeholder (researchers, practitioners and surgeons) roles are more equalised, and partnerships are emphasised [37]. The transformation to a rapid learning research system will require a concerted effort by research funders, academic institutions, healthcare systems, researchers, and a variety of practice, community, and policy stakeholders to evoke the culture shift in how people work and how research is co-created collaboratively.

There is help at hand, however. Multiple implementation science frameworks have increased potential for rapid uptake, such as: the Consolidated Framework for Implementation Research (CFIR) [50, 54], My Own Health Report (MOHR) [37], Practical, Robust, Implementation and Sustainability Model (PRISM) [40] and Reach, Effectiveness, Adoption, Implementation, and Maintenance (RE-AIM) [4, 40]. CFIR was used in the majority of the studies we reviewed [50, 54] and was an indirect focus by one other study [4]. CFIR is focused on components of system change and is intended to be flexible, so that researchers can tailor the framework to the specific intervention design, factors, and context being studied and is useful in guiding rapid-cycle evaluation of the implementation of practice transformation initiatives [50]. Gale et al. [50] carried out a rapid process evaluation guided by CFIR, completed within 12 months. Beck et al. [40] applied PRISM, combining it with RE-AIM components. The combination created a tailored implementation plan for Twenty-First Century well-child care, facilitating the implementation process and improving success in spreading and sustaining care models in paediatric practices. Battaglia and Glasgow [4] discussed RE-AIM as a framework for validating measures of reach, effectiveness, adoption, implementation, and maintenance, and raising the importance of sustainability as a key implementation outcome. Other examples applying rapid logic include MOHR [37], a practice-level, cluster randomized pragmatic implementation study designed to develop fast, actionable evidence around the use of patient-reported measures in patient care.

### Narrowing the gap - consequences leading to bridging the gap between research and practice

From the foregoing it is clearly important to bridge the gap between research and practice to ensure implementable interventions are current, relevant and applicable to real-time practice – encouraging uptake and ensuring it becomes established [4, 42, 50]. Randomised controlled trials (RCTs) are everywhere, but uncertainties exist in how rapid implementation could be addressed by the classic RCT [37, 52, 57]. Presently, evidence indicates that randomized efficacy trials take approximately five and a half years from the initiation of enrolment to publication, and seven years or more after adding in the time from grant application submission to enrolment initiation [53, 58, 61]. In the real-world environment of clinical practice, this time-lag is unacceptable, as well as impractical in a study on, say, a rare disease or the pandemic outbreak of COVID-19 [53, 58, 62]. An ethos of rapid implementation can help challenge the current static notion of good science (following a laborious, pipeline efficacy-effectiveness-implementation logic) where too much good science falls behind. The pipeline model suggests efficacy studies precede effectiveness or implementation research, and yet efficacy trials are often not relevant and are sometimes inversely related to those associated with success in later stages [11]. As a result, we often see a ‘voltage drop’ [4] (reduced fidelity of the intervention when disseminated to other settings), lack of guidance in tailoring interventions to the local context, and all-too-often, inadequate resources being made available for implementation. Clinical trials need to be more pragmatic; open to a range of methods, as necessary, to address a research question [11, 63], and able to address questions that are relevant to the multiple stakeholders involved. Comparing real-world alternatives, such as qualitative trials [10], basket trials or umbrella trials [43] can potentially be used as alternatives to the classic ‘placebo, no treatment, or control’. Bando [43] indicates, when considering drug testing, that it is important to have a trial design that can efficiently distribute targeted drugs and suggests umbrella and basket designs. Other authors such as Glasgow and Chambers [64] propose a blending of the efficacy and effectiveness stages of intervention development to improve the speed of knowledge creation and increase the usefulness and policy relevance of clinical research. Blending effectiveness and implementation research together has been referred to as the hybrid effectiveness-implementation typology [4, 11, 42]. The idea suggests rapid implementation research designs will hasten the movement of interventions from effectiveness testing through to implementation [62]. As proposed by Raine et al. [57] rapid implementation is moving past the classic large-scale multicentre Randomised Control Trials (RCTs) and towards implementing a broad menu of rapid methods. This evidence adds further support to our findings [10, 42, 43, 59, 60]. Our study should not be viewed as an endpoint, but as increasing understanding of rapid implementation and providing clarity for the next step in our field, that is, placing greater focus on applying and/or adapting rapid methods in implementation science and consideration on what future challenges and opportunities this may present.

### Phase 3; stage 2: strengths and limitations

To our knowledge, this new method combination (concept analysis and systematic integrative review) is introduced for the first time in this study, to assure analytical depth, rigour and replicability. However, by excluding non-English language studies, insights may remain limited. The process of applying the augmented three-phase framework that combined concept analysis with a systematic integrative review resulted in a robust process that enhanced the quality and transparency of the data produced. A clear conceptual definition of rapid implementation is now available and supports international work to implement more rapidly actionable results in clinical practice. Precision medicine is still in its infancy, but it holds the potential to fundamentally alter how evidence-based medicine is practiced and taught. We see the possibilities where rapid implementation meets precision medicine as potentially providing demonstrations needed at the intersection of precision medicine and rapid learning research systems. By way of finalising the discussion, we note the lack of research involving the patient’s voice, as well as the need to involve patients as additional stakeholders in implementation science research generally and particularly within rapid implementation. This points to the need for future research in this area [65].

## Conclusions

While rapid implementation is, in some ways, evolutionary, in other ways, it is revolutionary. There are new methods potentially dislodging current methods; longstanding theories and methods of doing research are being adapted and reconfigured, with many stakeholders (e.g., practitioners, patients, families, decision-makers, administrator and policy makers) recognising the need for faster answers to get results into practice more speedily, thereby negating criticisms of standard implementation delays and the research-practice gap. If it can be made to work, the future of rapid implementation informed by implementation science is bright. It can help efficiently integrate science into practice using contextual and systems perspectives, focusing on adaption, pragmatic trials and mixed methods, and engendering a degree of flexibility in data assessment and interpretation. The key remaining question is how far and fast can we go?

## Data Availability

Not applicable. All relevant data are within the article.

## Abbreviations

America: United States of America
CDSR: Cochrane Database of Systematic Reviews
CFIR: Consolidated Framework for Implementation Research
DARE: Database of Abstracts of Reviews of Effects
MOHR: My Own Health Report
PICO: P = population, I = phenomena of interest, Co = context
PRISM: Practical, Robust, Implementation and Sustainability Model
PRISMA: Preferred Reporting Items for Systematic Reviews and Meta Analyses
QAT: Quality Assessment Tool
QualSyst: Standard Quality Assessment Criteria for Evaluating Primary Research Papers
RE-AIM: Reach, Effectiveness, Adoption, Implementation, and Maintenance
RCTs: Randomised Control Trials
U.K.: United Kingdom
